# Social determinants of health influencing diabetes self-care behaviors among immigrants: a scoping review

**DOI:** 10.3389/fcdhc.2026.1915588

**Published:** 2026-09-01

**Authors:** Rie Kudoh, Taiga Shibayama

**Affiliations:** Institute of Medicine, University of Tsukuba, Ibaraki, Japan

**Keywords:** diabetes, immigrants, scoping review, self-care behaviors, social determinants of health

## Abstract

**Background:**

As globalization accelerates, immigrants face diabetes management challenges that are a public health concern. Although Social Determinants of Health (SDOH) are recognized as key drivers of health status and access to care, their role in diabetes self-care behaviors among immigrants remains unclear.

**Objectives:**

This scoping review aimed to identify and map SDOH influencing diabetes self-care behaviors among immigrants in host countries.

**Methods:**

PubMed and Web of Science were searched for peer-reviewed empirical studies (qualitative, quantitative, or mixed-methods) published in English between 2020 and 2024, following PRISMA-ScR framework, using terms related to immigrants, diabetes, self-care, and SDOH.

**Results:**

Twenty-five studies met inclusion criteria, predominantly from the North America and Caribbean region (n = 17), with no studies identified from South-East Asia or Africa. All six SDOH domains were associated with at least one of five self-care behaviors. Community and Social Context was the most pervasive domain, influencing all behaviors. Economic Stability, Health and Healthcare System, and Education Access and Quality were associated with multiple behaviors, with financial constraints and language barriers. Food Environment and Neighborhood and Physical Environment, as environmental factors, were more selectively associated; Food Environment with diet, and Neighborhood and Physical Environment with physical activity and medical visits.

**Conclusion:**

Diabetes self-care among immigrants is shaped by a complex interplay of structural and environmental factors, highlighting the need for culturally tailored, multilevel interventions to reduce self-care disparities. The absence of evidence from South-East Asia and Africa represents a critical gap, and future research in these underrepresented regions is warranted.

## 1 Introduction

The United Nations Department of Economic and Social Affairs defines an international Immigrant as “any person who changes his or her country of usual residence,” regardless of legal status, duration of stay, or reasons for moving (1). Immigrant population is a group of people from diverse cultural, socioeconomic and geographical backgrounds (2). The number of international migrants worldwide reached approximately 304 million in 2024, nearly doubling from 154 million in 1990 (3). Europe and Asia each hosted around 87 million and 86 million international migrants, respectively, comprising 61% of the global international migrant stock (4). These regions were followed by North America, with almost 59 million international migrants, accounting for 21% of the global migrant stock, Africa at 9%, Latin America and the Caribbean at 5%, and Oceania at 3% (4). Overall, the estimated number of international migrants has increased steadily over the past five decades since 1970, and approximately one in 30 people worldwide are migrants (4). Furthermore, an estimated 123.2 million people worldwide were forcibly displaced due to persecution, conflict, violence, human rights violations, and events seriously disturbing the public order in 2024, equating to 1 in every 67 people on Earth (5). Notably, displacement nearly doubled during the decade from 2015 to 2024 (5). One characteristic that warrants consideration in studies of immigrant health is the populations’ substantial heterogeneity (2). Immigrants often experience poorer health outcomes due to language and cultural barriers, institutional discrimination, and restrictive policies that limit access to or utilization of health services (1). Previous study has reported a higher risk of diabetes among foreign-born individuals compared to their native-born counterparts (2).

Diabetes is a chronic metabolic disease characterized by inflammation and hyperglycemia. It carries high mortality and morbidity risks due to long-term complications, including cardiovascular disease, diabetic retinopathy, and kidney failure (6). Diabetes poses a significant and growing global health burden, affecting approximately one in ten people worldwide (7). In addition to its rising prevalence, diabetes and its associated complications contributed to an estimated 6.7 million deaths among adults aged 20–79 years globally in 2021 (7). Three in four adults with type 2 diabetes live in low- and middle-income countries, and immigrant populations from low- and middle-income countries residing in high-income countries are particularly affected by type 2 diabetes and related complications compared with host populations (8). Evidence indicates that many immigrant groups have a higher prevalence of type 2 diabetes, develop the disease at a younger age, and experience poorer glycaemic control than native-born populations (9). Furthermore, studies have shown that foreign-born individuals have a higher overall prevalence of diabetes, including a substantial proportion of undiagnosed cases, compared with native-born populations (2).

A growing body of evidence indicates that social determinants of health (SDOH) play a critical role in diabetes. These determinants, including socioeconomic status, education, and access to healthcare, have been shown to influence not only the risk of developing diabetes but also disease management and clinical outcomes such as glycaemic control, cardiovascular risk factors, and quality of life (10). Given the high prevalence, substantial economic costs, and disproportionate population burden of diabetes, understanding and mitigating the impact of SDOH have been recognized as priorities, as reflected in the American Diabetes Association scientific statement on socioecological determinants of prediabetes and type 2 diabetes published in 2013 (11). Immigrants are particularly susceptible to the adverse effects of SDOH, as migration introduces additional structural and environmental barriers, including xenophobia, discrimination, language barriers, and limited access to health services (1). These factors collectively contribute to a disproportionate burden of chronic diseases, including diabetes, among immigrant populations. However, despite this evidence, the specific role of SDOH in shaping diabetes self-care behaviors among foreign-born residents remains poorly understood and insufficiently examined in existing literature. Thus, existing research in this area is fragmented across countries, and a comprehensive synthesis examining how various social factors interact to influence self-care behaviors in this population within each country’s specific healthcare and social context is lacking. The scoping review approach was chosen because this field remains insufficiently characterized in the existing literature, and a comprehensive mapping of the available evidence, regardless of study design, was considered more appropriate than a systematic review focused on a specific clinical question.

This scoping review aims to identify and map the SDOH influencing diabetes self-care behaviors among immigrants within the context of host countries’ healthcare systems and social environments. By systematically synthesizing the available literature, this study seeks to clarify how structural and environmental factors in receiving societies interact with individual self-care behaviors.

### 1.1 Definition of terms

Self-care behaviors:

In this study, self-care behaviors among patients with diabetes were operationalized across five domains: diet, physical activity, monitoring, medication adherence, and regular medical visits.

The rationale for this definition is as follows. Of the seven Self-Care Behaviors identified by the Association of Diabetes Care & Education Specialists (ADCES), Healthy Coping was excluded because, as Kent et al. (2010) noted, it represents a qualitative behavior that is difficult to quantify and is more appropriately conceptualized as a psychological prerequisite underlying concrete self-care behaviors rather than a discrete behavioral domain (12, 13). Similarly, Problem Solving involves cognitive processes that are not readily observable, and Reducing Risk encompasses a broad range of cognitive and behavioral dimensions oriented toward complication prevention. Given these characteristics, these three domains were deemed difficult to operationalize as quantitatively measurable daily behaviors. Accordingly, the present study adopted four foundational domains amenable to quantitative measurement: diet, physical activity, monitoring, and medication adherence. This approach is also consistent with the Japanese Diabetes Society’s Clinical Practice Guideline 2024, which identifies dietary therapy, exercise therapy, and pharmacotherapy as the core components of diabetes self-care, while conceptually distinguishing adherence and psychosocial issues as factors to be separately addressed when self-care behaviors are not functioning effectively (14).

Furthermore, the World Health Organization (WHO) defines self-care as “the ability of individuals, families, and communities to promote health, prevent disease, maintain health, and cope with illness and disability, with or without the support of a health worker” (15). The WHO Diabetes fact sheet further specifies that adverse diabetes outcomes can be avoided or delayed through diet, physical activity, and medication, in addition to regular screening and treatment of complications (6). Although regular medical visits may be interpreted as one component of Reducing Risk, they are characterized by active collaboration with healthcare providers and were therefore judged to warrant designation as an independent domain within the WHO conceptual framework outlined above. On this basis, regular medical visits were incorporated as the fifth self-care domain in the present study.

Social Determinants of Health (SDOH):

SDOH were defined in accordance with the framework proposed by Hill-Briggs (2020), comprising six domains: Economic Stability (income, employment, and housing security); Education Access and Quality (educational opportunities and health literacy); Community and Social Context (social relationships, cultural norms, and experiences of discrimination); Neighborhood and Physical Environment (living conditions and access to physical activity spaces); Health and Healthcare System (insurance coverage and availability of culturally competent providers); and Food Environment (availability and affordability of culturally appropriate nutritious food) (10).

## 2 Article types

Scoping Review articles

## 3 Method

A protocol for this review was not prospectively registered prior to data extraction, as this scoping review was conducted as an exploratory, preliminary investigation to map the existing literature on this emerging topic before undertaking a more formally registered systematic review. A systematic search was conducted across two databases: PubMed and Web of Science. Reporting followed the Preferred Reporting Items for Systematic Reviews and Meta-Analyses Extension for Scoping Reviews (PRISMA-ScR) guidelines (16). Search strategies are presented in **Table 1**. Furthermore, we searched the Web of Science using similar search criteria. The inclusion and exclusion criteria for article selection are shown in **Table 2**. No restrictions were placed on diabetes type or participant age; studies involving immigrants with any type of diabetes, including pediatric populations, were eligible if they reported associations between SDH and the defined self-care behaviors. The search was conducted in November 2024. The literature search and review were conducted independently by two reviewers (RK and TS). First, the title and abstract were assessed for eligibility. Next, full-text articles of relevant studies were reviewed. Discrepancy between the two reviewers was resolved by discussion. Data were charted using the following variables: study design, focus, sample size and population, outcomes, association between SDOH and self-care behavior, and study limitations. Data extraction was performed independently and in duplicate by the two reviewers (RK and TS) using a data charting form developed by the review team specifically for this study, in accordance with PRISMA-ScR guidance on data charting (16). During the initial phase of data extraction, after both reviewers had independently charted data from approximately 20 of the included studies, the extracted variables were reviewed and the data charting form was refined accordingly. Refinements included adding or removing specific data items, clarifying the classification criteria for the six SDOH domains, and refining the operational definition of the scope of “self-care behavior.” Discrepancies in extracted data and interpretation between the two reviewers were resolved through discussion across the full set of included studies.

**Table 1:** Search strategies for the three databases used in this study.

Databases (coverage dates)	Search strategies	Number of articles
PubMed	#1 immigrants OR emigrants OR transients OR migrants OR migration OR “Human Migration”[MeSH] #2 diabetes #3 self-management OR self-care #4 “Social Determinants of Health”[MeSH] OR “Sociological Factors”[MeSH] OR “Environment”[MeSH] OR “Environmental Exposure”[MeSH] OR “Food Supply”[MeSH] OR “Health Care Quality, Access, and Evaluation”[MeSH] OR “Costs and Cost Analysis”[MeSH] #5 #1 AND #2 AND #3 AND #4 #6 #1 AND #2 AND #3 AND #4 (Filters: last 5 years)	119
Web of Science	#1 TS= (diabet*) #2 TS= (immigrant* OR emigrant* OR transient* OR migrant* OR migration OR foreign* OR alien*) #3 TS= (self-management OR self-care) #4 TS= (“social determinant*” AND health) #5 TS= (socioeconomic* OR education* OR income* OR occupation*) #6 TS= (food AND (environment OR security OR insecurity OR access OR availability)) #7 TS= (neighborhood* OR housing OR “built environment*” OR “toxic environment*”) #8 TS= (“health care” AND (access OR affordability OR quality)) #9 TS= (social AND (context OR cohesion OR capital OR support)) #10 #4 OR #5 OR #6 OR #7 OR #8 OR #9 #11 #1 AND #2 AND #3 AND #10 #12 #1 AND #2 AND #3 AND #10 AND (2024 OR 2023 OR 2022 OR 2021 OR 2020)	167

**Table 2:** Inclusion and exclusion criteria for article selection.

Criteria	Inclusion	Exclusion
Time frame	Published between 2020 and 2024	Articles outside of the 2020–2024 time frame
Language	English language/Full text	Non-English language/Abstract
Place	Any host country where foreign residents’ healthcare experiences are documented	Nil
Research Focus	Peer-reviewed articles Involving immigrants diagnosed with diabetes Reporting social determinants of health (SDH) based on the Hill-Briggs (2020) framework, and their impact on the five defined self-care behaviors (Diet, Physical Activity, Monitoring, Medication Adherence, Regular Clinical Visits)	Full text not available Abstract not available Studies on short-term travelers or tourists (staying < 3 months). Studies focusing on gestational diabetes
Study Design	Original empirical research (qualitative, quantitative, or mixed-methods)	Literature reviews, systematic reviews, and meta-analyses (to focus on primary data). Interventional study Tool development Editorials, conference abstracts, case reports, and grey literature.

Extracted data were synthesized using a narrative, framework-based approach, in which reported associations between SDOH and self-care behaviors were mapped onto the six predefined SDOH domains and the five self-care behavior categories described above. Given the substantial heterogeneity in study designs, immigrant populations, and outcome measures across included studies, quantitative synthesis (e.g., meta-analysis) was not undertaken; instead, findings were qualitatively synthesized and organized within this SDOH-by-self-care-behavior matrix to identify recurring patterns, facilitators, and barriers across studies.

## 4 Results

**Figure 1:**
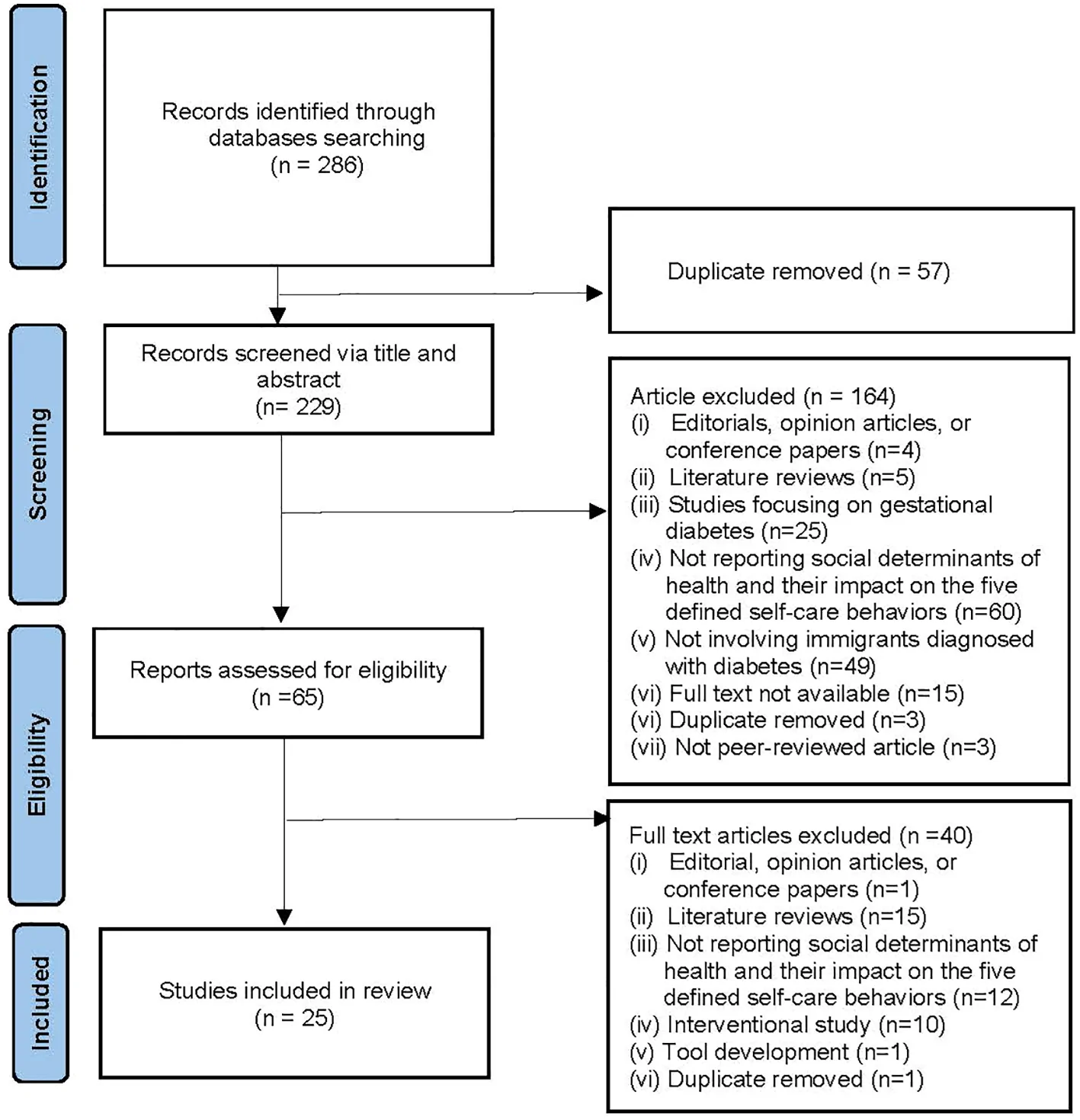
Flowchart of the article selection process. Template adapted from Page et al. (45).

The flowchart of the article selection process is shown in **Figure 1**. A total of 286 records were initially identified through database searching. After removing 57 duplicate records, 229 records remained and were screened via title and abstract. Screening led to the exclusion of 164 articles: editorials, opinion articles, or conference papers (n=4); literature reviews (n=5); studies on gestational diabetes (n=25); studies not reporting SDOH and their impact on the five defined self-care behaviors (n=60); studies not involving immigrants diagnosed with diabetes (n=49); studies with unavailable full text (n=15); duplicates (n=3); and non-peer-reviewed articles (n=3). The remaining 65 articles proceeded to full-text review for eligibility assessment. This full-text review led to the exclusion of 40 additional articles: editorials, opinion articles, or conference papers (n=1); literature reviews (n=15); studies not reporting SDOH and their impact on the five self-care behaviors (n=12); interventional studies (n=10); tool development studies (n=1); and duplicates (n=1). Finally, 25 studies met all inclusion criteria and were included in the analysis (17–41).

The distribution of studies by location is shown in **Table 3**, and study characteristics are summarized in **Table 4**.

**Table 3:** Distribution of studies by location.

Regional groups^†^	Country	Origin/ethnicity	Number of articles	Author (year)
North America and Caribbean; NAC (17)	United States	Iran, Turkey, and Lebanon	1	McConatha et al. (2020) (17)
		Haitian	2	Magny-Normilus et al. (2021) (20)
				Magny-Normilus et al. (2020) (41)
		Chinese	1	Niu et al. (2023) (25)
		Korean	1	Kim et al. (2023) (27)
		South Asian	1	Jamil et al. (2022) (21)
		Latinos	2	Titus et al. (2023) (33)
				Smith-Miller et al. (2022) (38)
		Mexican	1	Flórez et al. (2024) (28)
		Chinese or Filipino	1	Peregrina et al. (2024) (31)
		Immigrant or refugee born outside the US	1	Healy et al. (2024) (32)
		Nepali-speaking Bhutanese	1	Sharma et al. (2022) (37)
		Arab	1	El Masri et al. (2020) (40)
		Asian American and Pacific Islanders	1	Nguyen et al. (2022) (36)
	Canada	Black community	1	Swaleh et al. (2021) (19)
		Indonesian	1	Lukman (2023) (22)
		Somali	1	Deveci et al. (2024) (30)
South and Central America; SACA (1)	Dominican Republic	Haitian	1	Molina et al. (2023) (34)
Europe; EUR (3)	Norway	Turkish immigrants	1	Cokluk et al. (2023) (23)
	France	Migrants from Sub-Saharan Africa	1	de Monteynard et al. (2023) (24)
	Sweden	Migrants excluding those unable to understand Swedish, Arabic, Bosnian, or Spanish	1	Pettersson et al. (2023) (35)
Western Pacific; WP (2)	Australia	Sudanese	1	Roth et al. (2022) (18)
		Indian	1	Wan (2023) (26)
Middle-East and North Africa; MENA (1)	Saudi Arabia	Arabic-speaking immigrants	1	Althubyani, et al. (2024) (29)
South-East Asia; SEA (0)	None	None	None	None
Africa; AFR (0)	None	None	None	None
Two or more groups (1)	South Africa and Sweden	Ugandan	1	De Man et al. (2022) (39)

^†^ Regional groups: The International Diabetes Federation (IDF) is organised into seven regional groups.

**Table 4:** Overview of characteristics of the studies.

Author (year)	Study design	Focus	Sample size/population	Outcomes	Association between social determinants of health and self-care behavior		Limitation
					Diet× self-care behavior	Findings	
McConatha et al. (2020) (17)	A qualitative study	Cultural dimensions of diabetes management of Middle Eastern immigrants in the U.S.	28 older Middle Eastern immigrants who had been diagnosed with diabetes or pre-diabetes in the past 3 years	Wellbeing; social support; stress; life satisfaction; loneliness; lifestyle; illness management and coping; adaptation to diagnosis	Community and Social Context × Physical Activity	86% experienced increased stress attempting to increase physical activity 55% of men found it a major challenge 48% successfully increased activity regularly	Medical regimen management issues were not examined Small, non-representative sample (middle class, English-fluent, healthcare-accessible Middle Eastern participants) Community encapsulation and family relationship renegotiation were not addressed Based solely on patient interviews; potential under-disclosure of challenges
					Community and Social Context × Diet	90% reported dietary changes caused stress in relationships with family and friends, particularly around social gatherings 39% motivated by diagnosis to seek dietary support and reduce intake 80% of women preparing traditional family meals reported dietary changes as a continual source of stress 75% found social/familial gatherings less enjoyable due to dietary self-control 30% reported increased loneliness from reduced social engagement 60% found meal-centered gatherings stressful	
					Community and Social Context × All self-care domains	Family and social support was the strongest facilitator of successful lifestyle changes despite relationship stress	
Roth et al. (2022) (18)	A qualitative study	Knowledge and perceptions of type 2 diabetes self-management in a Sudanese community in Australia	12 Sudanese	Dinner plate drawing (visual dietary assessment); health literacy around diabetes and eye health; dietary management; exercise management	Community and Social Context × Physical Activity	Spousal and family encouragement were key facilitators of physical activity Family caregiving obligations (sick spouse, child with disability, childcare responsibilities) were major barriers to regular physical activity due to time constraints	Recruitment was difficult; more females than males were recruited, leaving perceptions of other gender groups unknown Language barriers may have limited depth of responses; some detail may have been lost Use of support persons as translators risked inaccurate interpretation, despite prior briefing by the researcher
					Community and Social Context × Diet	Family members reinforced provider dietary advice at home Social gatherings created pressure to eat non-recommended foods Ramadan fasting perceived as beneficial for blood glucose control and weight loss by most participants; however, fasting posed hypoglycemia risks for some, with doctors advising against it	
					Food Environment × Diet	Traditional Sudanese cuisine perceived as a major barrier; participants were advised by GPs and dieticians to reduce traditional staples	
					Education Access and Quality × All self-care domains	All participants understood the importance of healthy diet and physical activity for T2D management, demonstrating basic health literacy	
					Health and Healthcare System × Medical Visits	Language barriers impeded interactions with healthcare providers for non-English-fluent participants; some relied on family members for interpretation	
Swaleh et al. (2021) (19)	A qualitative study	Impact of health beliefs on diabetes self-management among Black Canadian adults	43 individuals in the Black Canadian community who either had a diagnosis of diabetes or were caring for someone with diabetes.	Diabetes management experiences; barriers to self-management; sources of social support; information-seeking behaviors (medications, diet, physical activity, complications); gaps in available resources	Economic Stability × Diet	High food costs led to greater carbohydrate consumption over vegetables among lower-income participants, resulting in poorer glycemic control	Geographically limited to the Greater Toronto Area Small sample size precluded stratification by age, socioeconomic status, education, or country of origin Unable to assess differences between heterogeneous subgroups (African, Caribbean immigrants and Canadian-born Black individuals)
					Health and Healthcare System × Diet	Culturally inappropriate dietary advice (focused on salads, pasta) excluded familiar foods (breadfruit, yam, sweet potato) Unfamiliar measurement systems created additional barriers to dietary adherence	
					Health and Healthcare System × Medication Adherence	Language barriers limited provider-patient discussion of treatment; short appointment times left patients frustrated and without adequate explanation Distrust of healthcare providers directly led to medication non-adherence	
Magny-Normilus et al. (2021) (20)	A qualitative study	Barriers to type 2 diabetes management among older Haitian immigrants	20 older adult Haitian immigrants age 65 years and older with type 2 diabetes	Demographic and clinical information (including self-reported A1C); diabetes self-management experiences	Economic Stability × Medication Adherence	Financial difficulties directly led to medication discontinuation; participants reported inability to afford insulin for months at a time	Qualitative design; findings are not intended for generalization Focused on northeast U.S. cities with established Haitian communities and churches for accessibility; barriers may differ in other populations Findings should be interpreted with caution due to geographic limitations
					Economic Stability × Diet	Economic hardship prevented purchase of healthy foods Cost was a direct barrier to dietary self-management	
					Community and Social Context × Diet	Church nurse support facilitated dietary changes and improved participants’ wellbeing	
Jamil et al. (2022) (21)	A qualitative study	Medication adherence and health beliefs among South Asian immigrants with diabetes in the U.S.	12 South Asian immigrants with cardiovascular disease and/or type 2 diabetes	Semi-structured interviews based on three frameworks (Dimensions of Medication Adherence, Patient Explanatory Model, HOPE questions); medication adherence assessed using modified Voils DOSE-Nonadherence measure	Community and Social Context × Medication Adherence	Family members reminded participants to take medications, purchased medications in emergencies, and facilitated communication with healthcare providers Open communication with family improved adherence	Sample limited to three South Asian subgroups (India, Pakistan, Bangladesh) speaking English, Urdu, or Hindi Participants were generally educated, medication-adherent, and acculturated; may not represent the broader South Asian immigrant population Modified adherence measure lacks validation in South Asian populations; self-reported adherence unconfirmed Social desirability and confirmation bias possible due to investigator’s dual role as pharmacist and South Asian background Limited community involvement hindered recruitment
					Health and Healthcare System × Medication Adherence	Effective provider-patient communication enabled participants to address side effects (e.g., adjusting or discontinuing medications) and achieve glycemic goals Established communication led to improved self-management outcomes	
Lukman (2023) (22)	A qualitative study	Religion, social support, and self-care experiences among Indonesian adults with chronic disease in Montreal	8 Indonesian immigrants who had a chronic disease	Living with illness experiences; meaning and practice of self-care; influences on self-care including religiosity and social support	Community and Social Context × All self-care domains	Spousal support encompassed daily activities, encouragement of healthy behaviors, stress management, and medication reminders Transnational family relationships provided emotional support, health advice, and access to traditional medicines from Indonesia	Single interviews with only 8 participants; findings may not be exhaustive, and repeated interviews or behavioral observation could have yielded greater depth Participants’ responses may have been filtered due to the researcher’s community membership Translation process risks information loss, potentially missing nuances in the data
					Community and Social Context × Monitoring	Spouses were the primary source of disease monitoring support Children assisted with blood pressure measurement	
					Community and Social Context × Physical Activity	Family members served as exercise partners Transnational family (abroad) encouraged exercise and monitored cardiovascular health remotely	
Cokluk et al. (2023) (23)	A qualitative study	Self-management of type 2 diabetes among Turkish immigrants in Norway	13 Turkish immigrants with type 2 diabetes	Self-management experiences of T2DM in relation to health literacy	Community and Social Context × All self-care domains	Cultural obligation to eat a host’s food was a dietary challenge; however, some participants developed confidence to decline food in social settings	Participants recruited primarily through mosques; religious lifestyle may influence findings and limit generalizability Data collected by a single researcher at one time point; may not capture changes in perspectives over time Interviews conducted in languages other than Norwegian or English relied on researcher translation, potentially losing exact participant wording
					Community and Social Context × Diet	Spousal support facilitated dietary management through daily meal preparation tailored to diabetes needs	
					Health and Healthcare System × Medication Adherence	Limited diabetes education at diagnosis (prescription given without structured education); participants were unaware of available diabetes education programs	
de Monteynard et al. (2023) (24)	A qualitative study	Perceptions of multimorbidity and coping strategies among Sub-Saharan African migrants with diabetes and HIV in France	12 Sub-Saharan African–born adults with HIV and type 2 diabetes from the Avicenne Hospital PLWHA cohort, on antidiabetic treatment, French-speaking	Disease discovery timeline; coping strategies (HIV and diabetes assessed separately); experience of managing multiple chronic conditions (polypathology)	Health and Healthcare System × All self-care domains	NGO support facilitated overall self-management for HIV; notably absent for diabetes in most interviews Religious beliefs served as a coping resource, reducing emotional distress around illness management across both conditions	Redundant question structure (HIV followed by diabetes) may have masked diabetes-specific challenges Single-center study; doctor–patient relationship models may not be representative Recruitment bias: most participants had controlled HIV and diabetes; findings may differ in less-controlled populations Only one insulin-treated participant; results largely limited to non-insulin-dependent diabetes Disease duration may have introduced memory bias
					Health and Healthcare System × Medication Adherence	Strong trust in medical providers was a key facilitator of medication adherence; positive experiences with HIV medication were transferred to diabetes medication management	
					Community and Social Context × All self-care domains	Family support eased the psychological burden of dual diagnosis and provided motivation to live; sharing diagnosis within family facilitated coping	
					Economic Stability × Diet	Financial constraints prevented purchase of vegetables and healthy foods, leading to consumption of non-recommended foods despite awareness of dietary guidelines	
Niu et al. (2023) (25)	Cross-sectional study	Prevalence and management of type 2 diabetes among Chinese Americans	Type 2 diabetes cases: 9,459 non-Hispanic Whites and 385 Chinese Americans	Diabetes management outcomes: medication use (insulin/pills), HbA1c testing frequency (≥2 times/year), daily glucose monitoring (≥1 time/day), eye examination (within 2 years), foot examination (≥1 time/year), receipt of provider-developed diabetes care, confidence in diabetes control	Community and Social Context × All self-care domains	LEP (OR = 0.18, 95% CI: 0.04–0.73) and first-generation status (OR = 0.25, 95% CI: 0.09–0.72) both associated with significantly lower confidence in diabetes control	Self-reported CHIS data subject to recall and reporting biases Data limited to California; findings may not generalize to other U.S. regions Small subgroup sample sizes and quasi-complete separation prevented intra-subgroup comparisons Low response rate
					Community and Social Context × Monitoring	LEP associated with significantly lower odds of daily blood glucose monitoring (OR = 0.17, 95% CI: 0.07–0.40); first-generation CAs also less likely to monitor daily than NHWs (OR = 0.22, 95% CI: 0.11–0.43)	
					Community and Social Context × Medical Visits	First-generation CAs had significantly lower odds of receiving provider-developed medical care (OR = 0.34, 95% CI: 0.18–0.68) English-proficient CAs more likely to have eye examinations than NHWs	
					Community and Social Context × Medication Adherence	Non-first-generation CAs had significantly higher odds of taking diabetes medications (OR = 3.21, 95% CI: 1.06–9.71)	
Wan (2023) (26)	Culturally concordant providers facilitated understanding of traditional dietary practices; provider feedback on glucose levels motivated self-management Lack of information about available diabetes support services in Australia at diagnosis was identified as a significant gap in care	Dietary management of type 2 diabetes mellitus among South Asian immigrants	18 first-generation Indian immigrants with type 2 diabetes, English/Hindi-speaking	24-hour dietary recall (3 non-consecutive days) as a proxy measure of dietary patterns	Community and Social Context × Diet	Indian community friends provided dietary advice after diagnosis; however, social pressure at gatherings also undermined dietary adherence Social obligations (frequent party invitations) were a major dietary barrier Some participants with diabetes withdrew from social contacts to maintain dietary control Differing food preferences within the family required cooking multiple separate meals, identified as the biggest dietary challenge	Self-reported dietary data subject to under- or over-reporting bias Traditional Indian dishes, herbs, and spices were absent from Australian food databases; proxy measures were used to minimize error Micronutrient data were not analyzed due to missing data from herbs and spices
					Health and Healthcare System × Diet	Culturally concordant providers facilitated understanding of traditional dietary practices; provider feedback on glucose levels motivated self-management	
					Health and Healthcare System × All self-care domains	Lack of information about available diabetes support services in Australia at diagnosis was identified as a significant gap in care	
					Health and Healthcare System × Medical Visits	Proactive appointment reminders from healthcare services facilitated preventive care and regular check-ups	
Kim et al. (2023) (27)	Cross-sectional study	Implications on self-care behaviors among older Korean immigrants diagnosed with diabetes residing in the U.S.	190 aged 55 or older Korean immigrants with diabetes	The Summary of Diabetes Self-Care Activities (SDSCA) questionnaire	Education Access and Quality × Medical Visits	Higher education level was a significant positive predictor of foot care (β=0.15, p<.05)	Online survey may have excluded older immigrants with limited digital access Small sample size limits generalizability SDSCA questionnaire did not include oral medication or insulin adherence items Regression coefficients were weak to moderate, possibly due to small sample size, outliers, or predictor variability
					Community and Social Context × Medical Visits	Family support was a significant negative predictor of foot care (β=−0.19, p<.05), suggesting greater family support may reduce individual initiative in preventive care	
Flórez et al. (2024) (28)	A mixed-methods design	Individual and network-level factors related to type 2 diabetes among Mexican American adults in New York City	81 self-identified Mexican American adults	Healthy Eating Index derived from 24-hour dietary recalls; International Physical Activity Questionnaire	Community and Social Context × Diet	Family had greater influence on home eating than friends Diabetic network members provided cautionary dietary advice despite struggling with their own self-management	Observational design precludes causal inference Non-probabilistic sample from a single NYC community; may not represent all Mexican Americans; data collected pre-COVID-19 limits current generalizability Single HbA1c measurement is atypical; confirmatory testing not performed Personal network data reflect only the ego’s perspective; discrepancies between ego and alter perceptions were not captured Mixed-method design limited the depth of qualitative inquiry
					Community and Social Context × Medical Visits	Medical doctors viewed as sole health authority despite social network support Some questioned validity of health information from network members	
					Community and Social Context × Medication Adherence	Stress impact on medication adherence recognized but difficult to convey to network members Spousal support facilitated clinic attendance	
Althubyani, et al. (2024) (29)	Cross-sectional study	Use of web-based technologies for diabetes self-management among Arabic-speaking immigrants with type 2 diabetes in Saudi Arabia	221 Arabic-speaking immigrants with type 2 diabetes	Disease history (T2DM duration, comorbidities); diabetes management difficulties (diet, physical activity, communication with healthcare providers, goal setting)	Education Access and Quality × Diet	Higher education significantly associated with intention to use web-based technology for dietary planning (OR = 3.30, p=0.039, 95% CI = 1.063–10.261)	Small convenience sample limits generalizability Survey not assessed for face validity in a culturally appropriate manner for Arabic-speaking immigrants, though the lead researcher’s cultural familiarity partially mitigated this
					Economic Stability × Medication Adherence	Approximately 48% of participants lacked health insurance, negatively impacting medication adherence	
					Community and Social Context × All self-care domains	Married participants were 4.9 times more likely to intend to use web-based technology, suggesting spousal support as a key facilitator Culturally and linguistically appropriate digital tools identified as important	
					Health and Healthcare System × Monitoring	Daily web-based technology use supported blood glucose monitoring 85% expressed intention to use web-based technology for dietary planning	
Deveci et al. (2024) (30)	A qualitative study	Perspectives on diabetes among Somali Canadian families and healthcare providers	Somali Canadian families of children with type 1 diabetes: 11 caregivers; 5 adolescents; 9 healthcare providers	Demographic questionnaire; A1C retrieved from medical charts; topics: perceived T1D control, treatment effectiveness, clinic satisfaction, cultural beliefs, management challenges, youth responsibility, future health impact, barriers and facilitators to care	Community and Social Context × All self-care domains	Canadian attitudes perceived to reduce children’s accountability and independence in self-management overall	Recent immigrants with limited English proficiency were not represented; their perspectives may differ from longer-term residents Small number of adolescent participants limits the range of experiences, though their HbA1c values were representative of the broader patient group
					Community and Social Context × Diet	Families adopted similar diets and eating schedules to accommodate the child’s diabetes management, reducing household flexibility Controlling food intake was the biggest perceived barrier to glycemic control, particularly in households with multiple children Adolescents struggled with dietary management in social situations Canadian food culture was perceived as contributing to difficulties with dietary management compared to life in Somalia	
Peregrina et al. (2024) (31)	A mixed-methods design	Family support in type 2 diabetes among older Chinese and Filipino American immigrants and their adult children	10 dyads of older Chinese and Filipino American immigrants with type 2 diabetes and adult children	Family roles before/after T2D diagnosis, T2D knowledge, management challenges, concerns about diagnosis/treatment, social interactions, and family social support experiences	Community and Social Context × Diet	Parents initially managed diet independently; adult children’s involvement gradually increased across four stages (Independence → Transitions → Partnership → Stepping In)	Small sample limits transferability to the broader Chinese and Filipino American populations with T2D Cross-sectional design captures caregiving dynamics at a single time point only
					Community and Social Context × Physical Activity	Parents initially managed physical activity independently; adult children’s support for exercise increased as stages progressed	
					Community and Social Context × Medical Visits	Adult children attended doctor visits only in the final “Stepping In” stage	
					Community and Social Context × Medication Adherence	Active support for medication adherence by adult children occurred only in the final “Stepping In” stage	
Healy et al. (2024) (32)	Cross-sectional study	Understanding patient and health care provider perceptions of barriers and facilitators to diabetes care in immigrant and refugee populations	19 immigrants/refugees; 35 healthcare providers	Barriers and perceived responsibility for diabetes care	Health and Healthcare System × All self-care domains	HCPs ranked culturally concordant care and cultural understanding as more important facilitators than immigrants/refugees did 44% of HCPs identified cultural sensitivity as the most important action Insurance coverage was the top barrier for HCPs Immigrants/refugees ranked it highest as a facilitator, suggesting shared recognition of its importance	No translation services or monetary incentives provided, likely reducing response rates and introducing sample bias toward those with higher health literacy and smaller language barriers Snowball sampling may have introduced social network bias Long U.S. residency and diabetes duration limit generalizability to recent immigrants and newly diagnosed patients Unknown comorbidities and inability to distinguish immigrant from refugee status were not assessed Diversity in opportunities, challenges, and privilege within the sample was not explored
					Education Access and Quality × All self-care domains	35.3% of immigrant/refugee patients wanted more diabetes education 36% of HCPs identified education provision as most important 23.5% of patients sought knowledge of latest treatments	
					Community and Social Context × All self-care domains	HCPs ranked peer/family support understanding diabetes management higher than patients Patients requested HCP empathy and citizenship assistance	
					Education Access and Quality × Medical Visits)	20% of HCPs identified appropriate language/translation services as the most important facilitator Language barriers were underestimated by HCPs	
Titus et al. (2023) (33)	A qualitative study	Immigrant Latinas Perspectives of Caring for their Diabetes	16 Latinas with type 2 diabetes	One question from the interview guide, “What do you do to take care of your health?”	Neighborhood and Physical Environment × Physical Activity	Agricultural fieldwork, cannery work, and household activities served as primary sources of physical activity	Snowball sampling may have limited findings to a select rural Latina population English fluency levels were unknown and may have affected participation in focus group discussions
					Food Environment × Diet	Abundance of locally grown vegetables supported healthy eating Home cooking perceived as healthier than street/fast food Working with a local dietician and avoiding high-fat/carbohydrate foods were effective strategies	
					Health and Healthcare System × Medication Adherence	All participants accessed medications through two local safety-net clinics or private pharmacies Spanish-speaking physicians and pharmacists available at both clinics facilitated adherence	
					Economic Stability × Diet	Higher U.S. income led to consumption of more expensive, perceived less nutritious foods Healthy foods were more affordable in Mexico Food costs in the U.S. were a barrier to healthy eating	
					Community and Social Context × All self-care domains	Intensive work culture after immigration led Latinas to neglect their own health Immigration itself was associated with dietary changes contributing to T2D	
Molina et al. (2023) (34)	A qualitative study	Enhancing diabetes health outcomes among Haitian migrants living in Dominican Republic	12 Healthcare providers providing care to adults with type 2 diabetes in a mobile clinic	Not reported	Economic Stability × All self-care domains	Extreme poverty and sole employment (sugarcane labor) perpetuated marginalization and negatively impacted T2DM management	Virtual data collection (Zoom) may have introduced distractions (e.g., unstable internet), potentially affecting responses Participants as FEUL members may have been predisposed to identifying barriers over facilitators Translation of Spanish data to English may have resulted in loss of nuances
					Economic Stability × Physical Activity & Medical Visits	Long working hours in sugarcane fields prevented clinic attendance among male participants	
					Food Environment × Diet	Little to no access to nutritious food and water severely limited dietary self-management	
					Health and Healthcare System × Medical Visits	No government healthcare benefits for Haitian migrants Limited prior experience accessing healthcare in either country	
					Health and Healthcare System × Medication Adherence	Lack of healthcare access and resources limited ability to obtain and adhere to prescribed medications	
					Community and Social Context × Medical Visits	Haitian cultural beliefs and distrust of Western medicine hindered adherence to provider recommendations	
					Community and Social Context × All self-care domains	Undocumented status and policies perpetuating social inequality were significant barriers to healthcare access	
Pettersson et al. (2023) (35)	A mixed-methods design	Self-care in migrants with type 2 diabetes, during the COVID-19 pandemic	79 migrants with type 2 diabetes, listed at a healthcare center	Self-Care of Diabetes Inventory (SCODI); self-care maintenance subscale	Health and Healthcare System × All self-care domains	Patients requested more information about COVID-19 and healthcare changes, as well as self-care tools (foot care materials, blood glucose monitors)	Cross-sectional design precludes causal inference Low response rate and written survey format likely excluded illiterate participants Given lower literacy rates among migrants, findings may not represent illiterate patients with T2D
					Health and Healthcare System × Medical Visits	76% reported changes in self-care during the pandemic; 58% changed active lifestyle maintenance and 40% changed physical exercise; some increased walking as a COVID-19 precaution	
					Neighborhood and Physical Environment × Physical Activity	25% experienced reduced healthcare access during the pandemic (fewer appointments, postponed care) Patients desired more check-ups, provider calls, and home visits for blood sugar testing	
					Food Environment × Diet	20% changed diet due to fatigue and inability to cook; conversely, some improved dietary control by avoiding impulse purchases and eating more regularly during the pandemic	
					Economic Stability × Medication Adherence	87% always took prescribed medications, suggesting relatively high medication adherence despite pandemic disruptions	
Nguyen et al. (2022) (36)	Cross-sectional study	Diabetes prevalence, risk factors, and care among Asian American and Pacific Islanders in Texas	Texas BRFSS adults: 34,961 non-Hispanic Whites; 3,739 Asian American and Pacific Islanders	Insulin use; blood glucose and A1C monitoring; diabetes health visits (annual foot exams, diabetes exam); diabetes management course attendance	Community and Social Context × All self-care domains	NHWs had significantly higher odds of insulin use, blood glucose monitoring, foot checks, and diabetes education course attendance than AAPIs	Unable to disaggregate data by individual AAPI ethnic subgroups, masking important health differences and limiting actionable interpretation Small subgroup sample sizes and inconsistencies in Texas BRFSS yearly questions precluded analysis of cholesterol, home blood pressure, family history, and e-cigarette use English/Spanish-only survey excluded older adults with lower health literacy or limited English proficiency, affecting validity of findings
					Community and Social Context × Monitoring	NHWs were 2.2 times more likely to check blood glucose daily than AAPIs	
					Economic Stability × Monitoring & Medication Adherence	Higher income (≥$75,000) associated with lower odds of insulin use, blood glucose monitoring, and foot checks vs. income <$25,000	
Sharma et al. (2022) (37)	A qualitative study	Influence of patriarchy on Nepali-speaking Bhutanese women’s diabetes self-management	15 female participants from the NSB community with type 2 diabetes	Demographic information (age, education, marital status, household size, language, religion); diabetes management; family dynamics; gender roles; cultural norms; healthcare access and autonomy; dietary practices; menstrual and religious fasting practices; health insurance knowledge	Education Access and Quality × All self-care domains	Poor health literacy and diabetes knowledge Skepticism toward western medicine despite provider explanations	Sample limited to NSB women in Harrisburg, PA; findings may not be generalizable to wider populations Virtual interviews due to COVID-19 could not ensure full participant privacy Gift card compensation may have introduced participation bias
					Education Access and Quality × Medical Visits	Limited language proficiency prevented independent appointment-making; unaware of healthcare rights	
					Community and Social Context × Medical Visits	Healthcare decisions made collectively by family Dependent on others for transportation to appointments	
					Community and Social Context × All self-care domains	Family members served as caretakers under PA waiver program; however, dependency on others was also observed	
					Community and Social Context × Medical Visits	Many participants visited traditional healers alongside regular doctors Ayurvedic and homeopathic treatments were also used as part of the healing process	
					Neighborhood and Physical Environment × Medical Visits	Lack of driving ability restricted healthcare access; women relied on family or community members for transportation	
					Food Environment × Diet	Rice central to cultural diet; white rice substituted with “bagada/diabetic” rice as perceived healthier alternative	
					Economic Stability × Physical Activity	Unemployed women more sedentary due to illness and reliance on family for household chores	
					Health and Healthcare System × Medication Adherence	Initial skepticism toward prescribed medications Reactive rather than preventive healthcare approach	
Smith-Miller et al. (2022) (38)	A mixed-methods design	Gender differences in type 2 diabetes self-management among Spanish-speaking Latinx immigrants	30 Spanish-speaking, limited English language proficiency, Latinx immigrant with type 2 diabetes	Health-Promoting Lifestyle Profile II (HPLP II); semi-structured interviews	Community and Social Context × Diet	Spouses/significant others actively supported dietary changes; spousal support was more prominent among male participants	Not reported
					Economic Stability × Diet	Economic constraints negatively impacted dietary choices; financial difficulties were more commonly reported by women	
					Economic Stability × Medical Visits & Medication Adherence	Economic constraints limited access to medical care and ability to purchase medications, particularly among women	
					Neighborhood and Physical Environment × Medical Visits	Women in rural areas faced additional barriers due to lack of public transportation and limited automobile access	
					Neighborhood and Physical Environment × Diet	Male manual laborers faced extreme working conditions limiting self-care; inadequate access to water led to consumption of sweetened drinks from food trucks	
De Man et al. (2022) (39)	Cross-sectional study	Motivational determinants of physical activity in disadvantaged populations with (pre) diabetes	Adults with prediabetes or diabetes: 712 Uganda, 566 South Africa, 147 Sweden	Treatment Self-Regulation Questionnaire; physical activity self-regulation	Education Access and Quality × Physical Activity	Educational level varied across samples; Ugandan participants reported highest vigorous PA despite lowest education level	Measure modifications across settings may have altered nuances due to translation and differing contextual relevance, limiting cross-setting comparability Sampling procedures differed across sites (random sampling in Uganda vs. health center recruitment elsewhere), reducing external validity Cross-sectional design precludes causal inference or assessment of trends over time
					Neighborhood and Physical Environment × Physical Activity	Ugandan and South African participants reported higher moderate PA compared to Swedish participants, suggesting environmental differences across settings influence PA levels	
					Community and Social Context × Physical Activity	Social support was significantly associated with PA outcomes across all three settings	
					Economic Stability × Physical Activity	Employment and marital status differed substantially across samples, potentially influencing PA behaviors	
El Masri et al. (2020) (40)	Cross-sectional study	Barriers and facilitators to perceived diabetes self-management in Arab American patients with diabetes	200 Arabic-speaking individuals or Arab Americans with type 2 diabetes	Diabetes self-management education and support in relation to Arab American culture	Community and Social Context × Diet	Family understanding of food choices was significantly associated with regular exercise (p=0.026) Receiving Arabic instructions significantly associated with food selection (OR = 6.66, p=0.014) and portion control (trend, OR = 3.67, p=0.066)	Majority of participants were Lebanese, who may not be representative of the broader Middle Eastern immigrant population given Lebanon’s multicultural and westernized characteristics Cross-sectional self-reported data subject to recall and social desirability bias, though mitigated by non-judgmental Arabic-speaking interviewers Survey not tested for reliability or validity
					Community and Social Context × Physical Activity	Family understanding of food choices remained significantly associated with regular exercise (OR = 2.41, p=0.03)	
					Community and Social Context × Medical Visits	Family encouragement significantly associated with discussion of care with a clinician (OR = 71.35, p=0.003) Time spent with family showed a trend association (p=0.066)	
					Education Access and Quality × Diet	Receiving instructions in Arabic significantly associated with food selection (OR = 6.66, p=0.014) and maintaining a healthy lifestyle (OR = 43.98, p=0.003); portion control showed a trend association (OR = 3.67, p=0.066)	
					Education Access and Quality × Physical Activity	Receiving instructions in Arabic significantly associated with regular exercise (p=0.021)	
Magny-Normilus et al. (2020) (41)	A qualitative study	Type 2 diabetes self-management among adult Haitian immigrants	16 Haitian immigrantswith type 2 diabetes, English- or Haitian Creole–speaking	Semi-structured interviews; topics: T2D diagnosis experience, typical day of self-management, post-diagnosis experiences; HbA1c, weight, and height retrieved from medical records; field notes recorded post-interview	Community and Social Context × All self-care domains	Despite strong family values, most participants managed T2D independently, with God as their primary daily support	Single geographic location limits generalizability Low male representation; gender diversity was insufficient to capture the full range of experiences in this population
					Community and Social Context × Monitoring	Blood glucose monitoring was inconsistent; most checked only when symptomatic (e.g., cold/clammy feeling, headaches, polyuria)	
					Neighborhood and Physical Environment × Diet & Physical Activity	Acculturation-related barriers (sedentary lifestyle, poor nutrition, limited resources) impacted self-management strategies	
					Food Environment × Diet	Differences in grocery shopping and cultural food beliefs guided dietary self-management strategies	
					Health and Healthcare System × All self-care domains	Most participants reported lack of cultural sensitivity from healthcare providers; desired better communication, respect, and culturally competent care	

### 4.1 Distribution of studies by location

A total of 25 studies were identified across six of the seven IDF regional groups; no studies were conducted in South-East Asia or Africa.

The majority were conducted in North America and the Caribbean (NAC, n = 17), predominantly in the United States (n = 16), examining diverse immigrant populations including Haitian (n = 2), Latino (n = 2), Chinese, and Korean groups. South and Central America (SACA) was represented by a single Dominican Republic study targeting Haitian immigrants. In Europe (EUR), three studies spanned Norway (Turkish immigrants), France (Sub-Saharan African migrants), and Sweden (multilingual migrants). The Western Pacific (WP) region contributed two Australian studies, focusing on Sudanese and Indian immigrant communities, respectively. The Middle East and North Africa (MENA) region was represented by one Saudi Arabian study examining Arabic-speaking immigrants. One additional study spanned multiple regional groups, involving participants from South Africa and Sweden focused on a Ugandan immigrant population.

### 4.2 Association between SDOH and self-care behavior

The following presents the associations between SDOH and self-care behaviors, organized according to five domains: diet, physical activity, medication adherence, monitoring, and medical visits.

#### Diet

Regarding diet, all six SDOH domains were identified as associated, with Community and Social Context the most frequently reported (17, 18, 20, 23, 26, 28–31, 38, 40). Family and spousal support facilitated dietary adherence through meal preparation tailored to diabetes needs (18, 23, 38) and cautionary advice from diabetic network members (28). Church nurse support facilitated dietary changes among Haitian immigrants (20). Conversely, social and cultural obligations at gatherings pressured non-recommended eating across multiple communities (17, 18, 26, 30). Among Middle Eastern immigrants, 90% reported dietary changes causing relationship stress, particularly around gatherings; 80% of women preparing traditional family meals described this as a continual stressor (17). Among South Asian immigrants, differing family food preferences required cooking multiple separate meals—the biggest dietary challenge—and some withdrew from social contacts to maintain control (26). Among Haitian immigrants in the U.S., despite strong family values, most managed diet independently (41). Family understanding of food choices was significantly associated with food selection among Arab Americans (40).

Economic Stability was a reported barrier (19, 20, 33): high food costs led to greater reliance on carbohydrates over vegetables (19, 20, 24), disproportionately affecting Latinx women immigrants (38). Health and Healthcare System influences included culturally inappropriate advice excluding familiar staples such as breadfruit, yam, and sweet potato (19), while culturally concordant providers facilitated understanding of traditional practices (26). Regarding Education Access and Quality, native-language diabetes instruction was associated with improved food selection and healthy lifestyle maintenance among Arab Americans (40); higher education was linked to intent to use web-based dietary planning tools (29).

Food Environment was both barrier and facilitator across six studies (17, 33–35, 37, 41). Providers advised against traditional cuisines, including Sudanese staples (18). Among Haitian migrants in the Dominican Republic, minimal access to nutritious food and water severely limited self-management (34). Among Nepali-speaking Bhutanese women, white rice—central to the cultural diet—was substituted with perceived healthier “diabetic” rice (37). Among Haitian Americans, grocery shopping behaviors and cultural food beliefs guided self-management strategies (41). Conversely, abundant locally grown vegetables and dietitian access supported healthy eating among rural Latina immigrants (33). Regarding Neighborhood and Physical Environment, acculturation-related barriers negatively affected self-management among Haitian immigrants (41), and male manual laborers’ inadequate access to safe water led to sweetened-beverage consumption from food trucks (38).

#### Physical activity

Regarding physical activity, Community and Social Context, Economic Stability, Education Access and Quality, and Neighborhood and Physical Environment were identified as associated SDOH domains (17, 18, 22, 31, 37, 39–41), with Community and Social Context the most frequently reported (17, 18, 22, 31, 39, 40). Family and spousal encouragement were key facilitators (18, 22), and social support was significantly associated with activity outcomes across all three settings in a multi-country study of Ugandan, South African, and Swedish participants (39). Family understanding of food choices was associated with regular exercise among Arab Americans (OR = 2.41, p = 0.03) (40). Adult children’s support for exercise increased progressively as caregiving stages advanced among Chinese and Filipino American families (31). Conversely, caregiving responsibilities, including care for a sick spouse or disabled child, were major time-related barriers (18). Among Middle Eastern immigrants, 86% reported increased stress when becoming more active, and 55% of men found it a major challenge (17).

Regarding Economic Stability, employment and marital status differed substantially across settings, potentially influencing activity levels (39). Among Nepali-speaking Bhutanese women, unemployment and illness led to more sedentary lifestyles with greater reliance on family for household chores (37). Regarding Education Access and Quality, Ugandan participants reported the highest vigorous activity despite the lowest educational level among the three populations (39). Native-language diabetes instruction was associated with regular exercise among Arab Americans (p = 0.021) (40). Regarding Neighborhood and Physical Environment, Ugandan and South African participants reported higher moderate activity than Swedish counterparts (39). Agricultural fieldwork, cannery work, and household activities served as primary activity sources among rural Latina immigrants (33). Acculturation-related barriers—including sedentary lifestyle and limited resource access—negatively affected physical activity self-management among Haitian immigrants in the U.S (41).

#### Monitoring

Regarding monitoring, Community and Social Context, Health and Healthcare System, and Economic Stability were identified as associated SDOH domains (22, 25, 29, 36, 41), with Community and Social Context the most frequently reported. Spousal support was a primary source of monitoring assistance, with spouses serving as the main support for disease monitoring and children assisting with blood pressure measurement among Indonesian immigrants (22). Blood glucose monitoring was frequently reactive rather than proactive; most Haitian immigrants checked glucose only when symptomatic, such as experiencing cold or clammy sensations, headaches, or polyuria (41). Language proficiency and generational status were significant determinants among Chinese Americans: limited English proficiency was associated with lower odds of daily monitoring (OR = 0.17, 95% CI: 0.07–0.40), and first-generation status showed a similar association (OR = 0.22, 95% CI: 0.11–0.43) (25). Non-Hispanic Whites were 2.2 times more likely to check blood glucose daily than Asian American and Pacific Islander participants (36). Regarding Economic Stability, higher income (≥$75,000) was paradoxically associated with lower odds of monitoring than income <$25,000 (36). Regarding Health and Healthcare System, daily use of web-based technology was identified as a facilitator of blood glucose monitoring (29).

#### Medication adherence

Regarding medication adherence, Community and Social Context, Economic Stability, and Health and Healthcare System were identified as associated SDOH domains (19–21, 24, 28, 29, 31, 33–36), with Community and Social Context the most frequently reported (21, 22, 25, 28, 31). Family members facilitated adherence through medication reminders, emergency procurement, and support for provider communication among South Asian immigrants (21). Spousal support encompassed medication reminders as part of daily self-management among Indonesian immigrants (22). Active support from adult children occurred in the final “Stepping In” caregiving stage among Chinese and Filipino American families (31). Non-first-generation status was significantly associated with higher odds of taking diabetes medications among Chinese Americans (OR = 3.21, 95% CI: 1.06–9.71) (25). Conversely, stress was a barrier to adherence that was difficult to convey to network members among Mexican Americans (28).

Regarding Economic Stability, financial difficulties directly led to medication discontinuation, including inability to afford insulin for months at a time among older Haitian immigrants (20). Approximately 48% of Arabic-speaking participants lacked health insurance, negatively affecting adherence (29). Economic constraints also limited medication purchases among Latinx immigrant women (38). Paradoxically, higher income (≥$75,000) was associated with lower odds of insulin use compared to income <$25,000 (36).

Regarding Health and Healthcare System, strong trust in providers was a key facilitator; positive experiences with HIV medication management transferred to diabetes adherence among Sub-Saharan African migrants in France (24). Effective provider-patient communication enabled participants to address side effects and achieve glycemic goals among South Asian immigrants (21). Access to Spanish-speaking physicians and pharmacists at safety-net clinics facilitated adherence among Latina immigrants (33). Conversely, limited diabetes education at diagnosis (23), language barriers limiting treatment discussion, short appointment times, and distrust of providers contributed to non-adherence among Black Canadians (19). Initial skepticism toward prescribed medications and a reactive approach to healthcare were also barriers among Nepali-speaking Bhutanese women (37). Among Haitian migrants in the Dominican Republic, limited healthcare access and extreme poverty further restricted medication adherence (34).

#### Regular medical visits

Regarding medical visits, Community and Social Context, Economic Stability, Health and Healthcare System, Education Access and Quality, and Neighborhood and Physical Environment were identified as associated SDOH domains (18, 25–28, 31, 32, 34, 35, 37, 38, 40).

Community and Social Context played a prominent role across multiple studies (25, 27, 28, 31, 34, 37, 40). Family encouragement was associated with discussing care with a clinician among Arab Americans (40). Adult children attended doctor visits only in the final “Stepping In” caregiving stage among Chinese and Filipino American families (31). Medical doctors were viewed as the sole health authority among Mexican Americans (28). Healthcare decisions were made collectively by family among Nepali-speaking Bhutanese women, whose dependence on others for transportation limited autonomous access; many also visited traditional healers and used Ayurvedic or homeopathic treatments alongside regular doctors (37). First-generation status was associated with lower odds of receiving provider-developed care among Chinese Americans, while English-proficient Chinese Americans were more likely to have eye examinations than non-Hispanic Whites (25). Haitian cultural beliefs and distrust of Western medicine hindered adherence to provider recommendations (34).

Regarding Economic Stability, long working hours in sugarcane fields prevented clinic attendance among male Haitian migrants in the Dominican Republic (34). Regarding Health and Healthcare System, language barriers impeded provider interactions, with some relying on family interpretation (18). Proactive appointment reminders facilitated preventive care among South Asian immigrants in Australia (26). During COVID-19, 76% of migrants in Sweden reported changes in self-care, and 25% experienced reduced healthcare access (35). Haitian migrants had no government healthcare benefits, and limited prior healthcare experience further restricted attendance (34).

Regarding Education Access and Quality, limited language proficiency prevented independent appointment-making and left Nepali-speaking Bhutanese women unaware of their healthcare rights (37). Among providers, 20% identified language and translation services as the most important access facilitator, though providers consistently underestimated language barriers relative to patients’ perspectives (32). Regarding Neighborhood and Physical Environment, rural Latinx women faced limited access to care due to inadequate public transportation (38), and Nepali-speaking Bhutanese women relied on family or community members for transportation due to limited driving ability (37).

### 4.3 Critical appraisal: study characteristics and reported limitations

As summarized in **Table 4**, the included studies varied in design, sample size, and population characteristics, predominantly employing qualitative or cross-sectional approaches. The included studies had several methodological limitations, limiting causal inference and generalizability. Qualitative studies relied predominantly on small, non-representative samples recruited through single institutions or snowball sampling, restricting transferability to broader immigrant populations. One study recruited participants primarily through mosques, limiting generalizability to those with different religious lifestyles (23). Among quantitative studies, low response rates and geographic restriction to single cities or regions raised selection bias concerns (25, 35, 36). Additional limitations included reliance on self-reported data subject to recall and social desirability bias, use of instruments not validated in target populations, and language barriers likely excluding participants with limited English proficiency (21, 25, 26, 29, 30, 32, 36, 40). Several studies also lacked sufficient within-group diversity to stratify findings by age, sex, socioeconomic status, or immigration status (19, 25, 36).

## 5 Discussion

This scoping review systematically mapped the SDOH associated with diabetes self-care behaviors among immigrants. The findings demonstrate that all six predefined SDOH domains were associated with at least one self-care behavior.

### 5.1 Distribution of studies by location

The geographic distribution of included studies revealed a concentration in the NAC region, particularly the United States, which accounted for more than half of the identified studies. North America alone hosted almost 59 million international migrants, 21% of the global migrant stock (4), which may partly explain this concentration. The limited representation of other regions suggests that the generalizability of current findings to immigrant populations elsewhere may be limited, given substantial differences in healthcare systems, social environments, and immigrant demographics across regions.

Of particular concern is the complete absence of studies from SEA and AFR, despite substantial migration in these regions. Asia hosted approximately 86 million international migrants, and Africa accounted for 9% of the global migrant stock (4). This lack of evidence represents a critical gap in the literature, as immigrants in South-East Asia and Africa may face distinct structural and environmental barriers to diabetes self-care not captured by existing research. Future studies in these underrepresented regions are needed for a more comprehensive understanding of how SDOH influence diabetes self-care across diverse global contexts, and to inform region-specific interventions responsive to the unique healthcare systems and social environments of these settings.

### 5.2 Association between SDOH and self-care behavior

#### Diet

Dietary self-care was associated with all six SDOH domains, most frequently Community and Social Context. Family and spousal support facilitated adherence, consistent with prior research on social networks and dietary behavior among ethnic minorities (42). Yet family support was not uniformly positive: social obligations at gatherings pressured non-recommended eating, and dietary changes sometimes strained relationships (17). This suggests family-centered interventions should address tensions between tradition and diabetes management. Economic Stability was a barrier in multiple communities, with high food costs linked to carbohydrate-heavy diets. Education alone is insufficient; affordable food access and financial support are equally critical, especially for women in economically marginalized communities (38). Health and Healthcare System factors showed a gap between practice and culturally appropriate care. Advice excluding familiar staples acted as a barrier (19), while culturally concordant providers facilitated self-management (26). Native-language instruction was significantly associated with improved dietary practices among Arab Americans (40), underscoring the value of linguistically and culturally tailored counseling. Food Environment was both barrier and facilitator across six studies (18, 33–35, 37, 41): limited access to nutritious food (34) and absent traditional cuisines in nutrition databases (18) created barriers, while local produce and dietitian access supported healthy eating elsewhere (33)—suggesting food environment effects depend on host-country context. Acculturation-related barriers in the Neighborhood and Physical Environment, including poor nutrition and limited resources post-immigration, were negatively associated with dietary self-management among Haitian immigrants (41), and unsafe water access was linked to sweetened-beverage consumption among male manual laborers (38). This indicates the physical environment itself can hinder dietary self-management beyond what individual or clinical interventions can address, pointing to a need for environmental and policy-level approaches (43, 44).

#### Physical activity

Regarding physical activity, Community and Social Context was the most prominent domain, with family and spousal encouragement facilitating activity across diverse immigrant communities (18, 22). However, caregiving responsibilities—such as caring for a sick spouse or child with a disability—posed significant time-related barriers (18), and 86% of Middle Eastern immigrants reported increased stress when attempting to become more active (17). This suggests physical activity interventions may benefit from family-based approaches that also address caregiving burdens disproportionately affecting immigrant populations. Regarding Economic Stability, unemployment and illness were associated with more sedentary lifestyles (37). Regarding Education Access and Quality, native-language diabetes education was significantly associated with regular exercise (40), supporting the value of linguistically appropriate health education in promoting physical activity. Built environment and occupational context also shaped activity levels: agricultural fieldwork and household tasks served as primary sources of physical activity in some communities (33), while acculturation-related barriers—including more sedentary routines and limited resource access—were negatively associated with physical activity among others (41), suggesting the transition to host-country environments may introduce new barriers to active living that warrant targeted intervention.

#### Monitoring

Community and Social Context played a significant role in shaping monitoring behaviors. While spousal and family support facilitated monitoring among Indonesian immigrants (22), language proficiency and generational status emerged as critical determinants: limited English proficiency and first-generation status were significantly associated with reduced monitoring frequency among Chinese Americans (25), and Non-Hispanic Whites were 2.2 times more likely to monitor blood glucose daily than Asian American and Pacific Islander participants (36). This suggests first-generation immigrants and those with limited English proficiency may be at particular risk of inadequate monitoring, highlighting the need for targeted support for this subgroup. A notable finding was that monitoring behaviors were often reactive rather than proactive, with most Haitian immigrants checking glucose only when symptomatic (41)—possibly reflecting insufficient diabetes education at diagnosis or limited awareness of routine monitoring’s importance. This suggests targeted education emphasizing proactive self-monitoring may benefit immigrant populations who often face barriers to diabetes information and educational resources. The paradoxical finding that higher income was associated with lower odds of blood glucose monitoring (36) warrants further investigation. Finally, web-based technology was identified as a facilitator of blood glucose monitoring (29), pointing to the potential of digital health interventions as an accessible, scalable approach to supporting monitoring among immigrant populations—provided that language-accessible and culturally appropriate resources are developed.

#### Medication adherence

Community and Social Context was the most frequently reported domain regarding medication adherence, with family support facilitating adherence through medication reminders, emergency procurement, and provider communication across multiple immigrant communities (21, 22). The association between non-first-generation status and higher odds of taking diabetes medications among Chinese Americans (25) suggests that greater familiarity with host country healthcare systems through acculturation may facilitate adherence, paralleling findings observed for monitoring behaviors. Economic Stability emerged as a critical structural barrier, with financial difficulties directly associated with medication discontinuation among older Haitian immigrants (20), and approximately 48% of Arabic-speaking participants lacking health insurance, which was negatively associated with adherence (29). This suggests medication adherence cannot be addressed through behavioral interventions alone, and that structural solutions—including expanded health insurance coverage and medication subsidy programs—are needed (10). The paradoxical finding that higher income was associated with lower odds of insulin use (36), consistent with the pattern observed for blood glucose monitoring, warrants further investigation. Health and Healthcare System factors revealed the importance of provider-patient relationships in shaping adherence. Strong provider trust and effective communication facilitated adherence (21, 24), while language barriers, limited diabetes education at diagnosis, short appointment times, and cultural distrust of Western medicine contributed to non-adherence across multiple communities (19, 23). These findings suggest that improving adherence may require not only structural changes such as extended appointment times and language-accessible services, but also sustained efforts to build provider-patient trust through culturally responsive care, with interventions tailored to each group’s specific cultural beliefs and healthcare experiences.

#### Regular medical visits

Regarding medical visit, Community and Social Context played a particularly prominent role, with family support acting in both facilitating and paradoxical ways. Family encouragement was strongly associated with clinician engagement among Arab Americans (40), yet collective family decision-making limited autonomous healthcare access among Nepali-speaking Bhutanese women (37). The association between first-generation status and lower odds of receiving provider-developed care among Chinese Americans (25) mirrors patterns seen in medication adherence and monitoring, suggesting unfamiliarity with host-country healthcare systems is a cross-cutting barrier across self-care behaviors. The concurrent use of traditional healers alongside Western medicine in several communities (34, 37) further underscores the need to respect diverse health belief systems when designing attendance interventions.

Health and Healthcare System factors revealed both barriers and facilitators. Proactive appointment reminders supported preventive care among South Asian immigrants (26), while language barriers and absent government healthcare benefits limited attendance elsewhere (18, 34). This suggests host-country healthcare systems can either enable or impede attendance, and provider-initiated support—such as reminders and follow-up contact—may be more effective than patient-initiated care alone for populations facing structural access barriers.

Regarding Education Access and Quality, providers consistently underestimated language barriers relative to patients’ actual experiences (32), suggesting existing language services may not meet patients’ real needs. Limited language proficiency impeded appointment-making and left patients unaware of their healthcare rights (37), highlighting the need for providers to actively inform patients of available services and entitlements alongside language support. Neighborhood and Physical Environment barriers—particularly limited transportation—disproportionately affected women in rural communities (37, 38), reflecting geographic and gender-based health inequities, addressable through structural solutions such as community transportation support and telehealth services rather than individual-level interventions alone.

Across these five self-care behaviors, several SDOH did not operate in isolation but exerted cross-cutting influences on multiple domains of self-care simultaneously. Language barriers emerged as a recurring determinant, affecting not only communication during medical visits (18, 32, 34, 37) but also comprehension of dietary counseling (40), engagement in physical activity education (40), and frequency of glucose monitoring (25). Acculturation and generational status likewise showed a consistent pattern across behaviors: first-generation and less-acculturated immigrants demonstrated reduced monitoring frequency (25), lower rates of provider-developed care (25), and greater exposure to environmental barriers to diet and physical activity following immigration (41), while non-first-generation status was associated with higher medication adherence (25). This suggests that unfamiliarity with host-country healthcare systems and cultural norms constitutes a shared underlying barrier across self-care behaviors, rather than a domain-specific issue. Similarly, economic disadvantage constrained multiple aspects of self-care concurrently, including food affordability (38), time and capacity for physical activity (37), and access to medications and insurance coverage (20, 29), indicating that financial hardship may compound barriers across behavioral domains rather than affecting a single behavior in isolation. Finally, the quality of the patient-provider relationship, shaped by cultural concordance, trust, and communication, appeared to influence dietary counseling (26), medication adherence (21, 24), and attendance at medical visits (26, 34, 37) alike. Taken together, these findings suggest that SDOH interact synergistically rather than independently: an immigrant facing language barriers, limited acculturation, and economic disadvantage is likely to experience compounded difficulties across several self-care behaviors simultaneously, rather than an isolated barrier in a single domain. This underscores the value of multicomponent interventions that address several SDOH domains concurrently—such as combining language-accessible services, culturally concordant care, and economic support—rather than single-domain approaches targeting one behavior or one determinant at a time.

### 5.3 Implications for practice and future research

The findings of this scoping review highlight several implications for diabetes self-care among immigrant populations in host countries. From a clinical practice perspective, the integration of culturally concordant care into diabetes management services emerged as a key strategy for improving self-management outcomes across multiple self-care behaviors (26, 32, 40). For healthcare facilities serving diverse immigrant populations, establishing language-accessible services — including interpreter support and native-language educational materials — would contribute to reducing communication barriers between providers and patients (18, 19, 23, 32, 37, 40). The systematic discrepancy identified between healthcare providers’ and patients’ perceptions of language barriers, with language barriers consistently underestimated by healthcare providers, underscores the importance of cultural competency training as a core component of provider education (32). In the context of community-based care, peer support networks, and community health workers may serve as valuable resources for delivering culturally tailored self-management interventions, given the prominent role of Community and Social Context identified across all five self-care behaviors. Furthermore, addressing structural inequities, including lack of health insurance coverage, inadequate public transportation infrastructure, and occupational marginalization, through policy-level interventions may contribute to reducing disparities in diabetes self-management outcomes among immigrant communities (29, 32–34, 37, 38). While the findings of this review do not allow definitive conclusions regarding which SDOH domain exerts the greatest influence on self-care, the identification of domain-specific associations across five self-care behaviors may provide a useful framework for prioritizing intervention targets. These findings may also offer valuable insights for policymakers and healthcare providers in countries experiencing increasing immigrant populations, where culturally responsive diabetes care remains an evolving challenge.

From a research perspective, future studies would benefit from adopting prospective, longitudinal designs that can clarify the temporal and causal relationships between SDOH and diabetes self-care behaviors among immigrant populations, moving beyond the cross-sectional designs that characterized most included studies. In addition, intervention-based research—including pilot studies and randomized controlled trials of culturally tailored, multicomponent interventions addressing the cross-cutting SDOH identified in this review, such as language-accessible education, culturally concordant care, and structural support for economic and healthcare access—is needed to establish causal evidence for strategies that can effectively improve diabetes self-care in these populations. Comparative studies explicitly contrasting different immigrant groups, for example, across migration pathways, legal status, generational status, or geographic regions, would also help distinguish SDOH that operate universally across immigrant populations from those that are specific to particular cultural, legal, or migration contexts, thereby informing more precisely targeted and culturally appropriate strategies. Moreover, larger-scale, multi-site studies with culturally adapted and validated outcome measures, incorporating native-language data collection, would be needed to improve the generalizability of findings and ensure adequate representation of underserved immigrant subgroups. Additionally, research examining the cost-effectiveness of culturally tailored interventions and their impact on health equity across diverse geographic and immigration contexts may contribute to a more comprehensive evidence base.

It should also be noted that the immigrant populations included in this review differed not only in country of origin but also in migration pathway and legal status, which may limit the transferability of findings across immigrant groups. For instance, Molina and Enriquez’s study of Haitian migrant laborers in the Dominican Republic (34) documented barriers rooted in undocumented status and the absence of government healthcare benefits—barriers of a fundamentally different nature and severity than those reported among more established immigrant communities, such as language or cultural barriers described among Chinese American (25) or Arab American (40) populations with more secure legal status. Similarly, populations with refugee or asylum-related migration histories (24, 35) may face distinct combinations of trauma-related, legal, and resettlement-related barriers not captured among labor or family-reunification migrants. These differences suggest that findings regarding facilitators such as family support or culturally concordant care, largely derived from settled immigrant communities, may not directly generalize to populations facing precarious legal status or recent forced displacement. Future studies would benefit from explicitly reporting migration pathway and legal status alongside country of origin, as these factors may independently shape both exposure to SDOH and access to diabetes self-care resources.

While no age restrictions were applied during study selection, the vast majority of included studies (24 of 25) focused on adult immigrant populations, predominantly with type 2 diabetes. Only one study specifically addressed a pediatric/adolescent population, examining Somali Canadian adolescents with type 1 diabetes and their families (30). Notably, this study revealed a caregiving pattern distinct from that observed among adult populations: parents and caregivers served as the primary agents of diabetes management for adolescents, reflecting the developmental dependency characteristic of pediatric chronic disease care. This contrasts with the pattern observed among older adult immigrants, where adult children increasingly assumed caregiving roles as their parents’ self-management needs evolved (31), illustrating a reversal in family caregiving direction across the life course. These findings suggest that self-care behaviors, family involvement, and the relevance of specific SDOH domains may differ considerably between pediatric and adult immigrant populations—for instance, school environments and parental health literacy may be more salient SDOH for children with diabetes, whereas occupational and healthcare system factors may be more relevant for working-age adults. Given the limited representation of pediatric evidence in this review, findings regarding family and community support should be interpreted primarily within the context of adult self-care. Future reviews and primary studies with sufficient pediatric samples are needed to enable age-stratified synthesis and to clarify how SDOH operate differently across developmental stages among immigrant populations with diabetes.

### 5.4 Limitations

This scoping review has several limitations. First, the literature search was restricted to two databases, PubMed and Web of Science, which may have resulted in the omission of relevant studies indexed elsewhere; these two databases were selected for their broad, multidisciplinary coverage of both health and social science literature relevant to SDOH, though future reviews may benefit from including additional databases. Second, the search was limited to English-language studies, potentially introducing language bias and excluding relevant non-English literature; this restriction was applied to ensure consistent interpretation of study findings without the risk of translation-related errors. Third, the search was limited to studies published between 2020 and 2024, so older but potentially relevant literature was not captured; this timeframe was deliberately chosen to prioritize the most recent evidence reflecting current healthcare systems and immigration contexts. Fourth, no protocol was prospectively registered prior to data extraction; this review was conducted as an exploratory investigation to map existing literature in this emerging area, and the absence of registration may introduce reporting bias and potential for *post-hoc* decisions in study selection or data extraction; to mitigate this risk, study selection and data extraction were conducted independently and in duplicate by two reviewers throughout all stages, with discrepancies resolved through discussion. Fifth, the geographic distribution of included studies was heavily skewed toward the North America and Caribbean region (n = 17), with no studies identified from South-East Asia or Africa, limiting generalizability to immigrant populations in these regions; as this gap reflects an absence of evidence rather than a methodological choice, it could not be mitigated within the current review, and we instead highlight it as a priority for future primary research. Sixth, heterogeneity in study designs, immigrant populations, and outcome measures across included studies precluded quantitative synthesis, warranting cautious interpretation of findings; to address this, findings were synthesized using a structured, framework-based narrative approach to enable systematic comparison across studies despite this heterogeneity. Seventh, although no age restrictions were applied, the included studies were overwhelmingly focused on adult populations, with only one study addressing a pediatric/adolescent population (30); this imbalance limits our ability to draw robust conclusions regarding age-related differences in SDOH and self-care behaviors, and we have accordingly interpreted findings on family and community support primarily within the context of adult self-care. Finally, as a scoping review, this study aimed to map existing evidence rather than assess individual study quality; consistent with PRISMA-ScR guidance, which does not require formal quality appraisal for scoping reviews, methodological limitations inherent in the included studies—such as reliance on self-reported data, small sample sizes, and cross-sectional designs— were documented and summarized to provide readers with context for interpreting the certainty of the evidence.

## 6 Conclusion

This review identified SDOH affecting diabetes self-care (diet, physical activity, medication adherence, monitoring, medical visits) among immigrants. Community and Social Context was the most influential domain: family support facilitated self-management, while cultural obligations, caregiving duties, and acculturation stress acted as barriers. Economic Stability, Healthcare System, and Education domains were linked to multiple behaviors, mainly via financial and language barriers. Food and Neighborhood Environments showed more selective associations with diet and physical activity, respectively.

These effects were not uniformly negative—culturally concordant care, native-language education, family support, and safety-net healthcare access also facilitated self-management, though unevenly across communities, reflecting structural inequities in host-country systems.

Findings call for culturally tailored, multilevel interventions prioritizing language-accessible services and community-based support. A key limitation is the absence of studies from South-East Asia and Africa, despite Asia being a major migrant-origin region; future research there is needed for a more globally applicable understanding of SDOH in diabetes self-management.

## Data Availability

The original contributions presented in the study are included in the article/supplementary material. Further inquiries can be directed to the corresponding author.
